# Parliament and the Profession

**Published:** 1916-07-01

**Authors:** 


### PARLIAMENT AND THE PROFESSION.

### The Campaign against Tuberculosis.

On the first day of the reassembly of the House of Commons there was a debate on the vote for administration of the Insurance Act. Mr. Charles Roberts pointed out that war conditions made it impossible to deal administratively with a number of questions that were too controversial to touch at the present time. For instance, it had been suggested that something might be done with regard to sanatorium treatment or with questions connected with the payment of doctors, but these things must wait until the return to normal conditions. In the course of his lengthy statement, Mr. Roberts made special reference to the treatment of tuberculosis. With the money which had been available, both through the Insurance Act and by means of a grant, the country had been covered with a network of hospitals, sanatoria, and dispensaries, and in connection with that and by voluntary effort and otherwise there had been tuberculosis schools and care committees set up. Mr. Roberts then proceeded to explain the progress that had been made in connection with the treatment of tuberculosis, dealing with the year 1914, which was the only year for which he had full information. The applications for treatment that year increased by 6 per cent., the persons treated increased 17 per cent., the cases treated in residential institutions increased 69 per cent., and the cases treated in dispensaries increased 232 per cent. In 1915 the dispensary treatment went up to a very large percentage; the treatment in residential institutions dropped by a small percentage—a percentage not greater than the reduction in the insured population during the year. Tuberculous soldiers had been treated with special attention; the Commissioners had had 3,250 cases mentioned to them by the War Office, and he believed treatment had been offered in residential institutions for all these cases. He quite admitted that they had not as large a fund as he should like to have; still, under the Insurance Act they had found, since it had been in force, the sum of £2,800,000. It had been spent in an attack on tuberculosis by means of sanatorium benefit, and, so far from that fund drying up, insurance committees in 1916-17 would have an income of £760,000, including a grant of £35,000 for tuberculous soldiers, so that the income for that year would be a considerable sum. When the war was over he hoped to see a still larger expenditure of money in eradicating, as far as could be done, tuberculosis from the country.

### Petrol for Doctors' Cars.

In Committee on the Finance Bill Mr. Montagu gave some information as to the nature of the control which the Petrol Control Committee intends to institute over the petrol stocks. The Committee having obtained from consumers the average of their past consumption, and their estimate of their future consumption, will issue a permit to each customer entitling him to purchase a specified number of gallons of petrol for a specified purpose and within a specified period. In order to get that permit from the Petrol Control Committee the consumer will pay a licence duty of 6d. for every gallon which the permit enables him to obtain. If he does not take out the quantity of petrol during the time specified he will be entitled to a refund. Commercial cars, doctors' cars, and the cars of veterinary surgeons will be entitled to a permit at half-rates. At the conclusion of Mr. Montagu's statement, Mr. Rutherford asked whether this was to be in the place of the old tax, to which Mr. Montagu replied: "Oh, no, no! The old tax will go on. This is a new duty on the licence to buy petrol."

